# Evaluation of the Possible Pathways Involved in the Protective Effects of Quercetin, Naringenin, and Rutin at the Gene, Protein and miRNA Levels Using In-Silico Multidimensional Data Analysis

**DOI:** 10.3390/molecules28134904

**Published:** 2023-06-21

**Authors:** Seema Zargar, Nojood Altwaijry, Tanveer A. Wani, Hamad M. Alkahtani

**Affiliations:** 1 Department of Biochemistry, College of Science, King Saud University, Riyadh 11495, Saudi Arabia; nojood@ksu.edu.sa; 2 Department of Pharmaceutical Chemistry, College of Pharmacy, King Saud University, Riyadh 11451, Saudi Arabia; twani@ksu.edu.sa (T.A.W.); ahamad@ksu.edu.sa (H.M.A.)

**Keywords:** flavonoids, gene interactions, computational analysis, pathways, miRNAs

## Abstract

Flavonoids are secondary metabolites that are non-essential for plant growth or survival, and they also provide numerous health benefits to humans. They are antioxidants that shield plants from the ill effects of ultraviolet light, pests, and diseases. They are beneficial to health for several reasons, including lowering inflammation, boosting cardiovascular health, and lowering cancer risk. This study looked into the physicochemical features of these substances to determine the potential pharmacological pathways involved in their protective actions. Potential targets responsible for the protective effects of quercetin, naringenin, and rutin were identified with SwissADME. The associated biological processes and protein–protein networks were analyzed by using the GeneMANIA, Metascape, and STRING servers. All the flavonoids were predicted to be orally bioavailable, with more than 90% targets as enzymes, including kinases and lyases, and with common targets such as NOS2, CASP3, CASP9, CAT, BCL2, TNF, and HMOX1. TNF was shown to be a major target in over 250 interactions. To extract the “biological meanings” from the MCODE networks’ constituent parts, a GO enrichment analysis was performed on each one. The most important transcription factors in gene regulation were RELA, NFKB1, PPARG, and SP1. Treatment with quercetin, naringenin, or rutin increased the expression and interaction of the microRNAs’ hsa-miR-34a-5p, hsa-miR-30b-5p, hsa-let-7a-5p, and hsa-miR-26a-1-3p. The anticancer effects of hsa-miR-34a-5p have been experimentally confirmed. It also plays a critical role in controlling other cancer-related processes such as cell proliferation, apoptosis, EMT, and metastasis. This study’s findings might lead to a deeper comprehension of the mechanisms responsible for flavonoids’ protective effects and could present new avenues for exploration.

## 1. Introduction

Quercetin, naringenin, and rutin have antioxidant capacities, thus preventing cell injury by free radicals [1,2,3]. The anti-inflammatory properties possessed by them can result in a reduced production of inflammation-causing products [4]. Furthermore, evidence suggests that they may have anticancer [5] capabilities, specifically targeting cancer cells by decreasing their viability and triggering their apoptosis. Flavonoids have neuroprotective properties and may be helpful in neurodegenerative disorders such as Alzheimer’s or Parkinson’s [6].

Despite these promising findings, much more research into the pharmacological properties of these flavonoids is necessary. This entails investigations into their drug-like properties, drug targets (genes or proteins), and mechanisms of action. They can modulate the expression or activity of various genes or proteins involved in cellular processes such as inflammation, oxidative stress, cell cycle regulation, apoptosis, and angiogenesis [7,8]. Some examples of gene or protein targets for quercetin, naringenin, and rutin are NF-κB, AP-1, Nrf2, COX-2, iNOS, TNF-α, IL-1β, IL-6, IL-8, MAPKs, PI3K/Akt, JAK/STAT, caspases, Bcl-2 family, p53, cyclins, CDKs12, and VEGF [7,8,9,10,11]. Polyphenols have a complicated and multi-faceted mode of action. The polyphenols may neutralize free radicals and chelate metal ions, making them useful antioxidants [12]. They can regulate gene expression and protein function by interacting with receptors, kinases, or transcription factors, which, in turn, alter the resulting signaling pathways [13,14,15,16,17]. They can also affect cell cycle progression and apoptosis by modifying the expression or activity of cyclins, CDKs, caspases, and Bcl-2 family members. The inhibition of angiogenic VEGF expression or activity by polyphenols is another mechanism by which they work [18,19].

The potential health advantages can be better understood by using computational tools to study and simulate the biological processes [20]. The use of computational models has allowed scientists to test hypotheses and create accurate predictions about the biological effects of flavonoids, such as their possible anticancer, antioxidant, anti-inflammatory, cardiovascular, and anti-neurotoxicity effects.

The liver is responsible for metabolizing quercetin, naringenin, and rutin, all of which are absorbed via the small intestine. Transporters such as OATP1A2, OATP2B1, and MRP2 help facilitate the absorption of the studied drugs. UDP-glucuronosyltransferases (UGTs), sulfotransferases (SULTs), and catechol-*O*-methyltransferases (COMTs) play important roles in the extensive metabolism of these substances after absorption [21]. Genes and transcription factors that control lipid metabolism and oxidative stress have been demonstrated to be activated by rutin in several investigations [13,14]. Specifically, rutin upregulates PPAR-alpha, a transcription factor that regulates lipid metabolism. Additionally, rutin, like quercetin, stimulates Nrf2, which is a major factor in controlling oxidative stress [22]. In other investigations, breast cancer cells were treated with naringenin and then analyzed to see which genes were differently expressed. These data showed that naringenin increased the expression of genes that work to suppress cell growth, inflammation, and blood vessel formation [23]. Studies have revealed that genes and transcription factors are activated in response to quercetin, naringenin, and rutin, which may account for their protective effects [15,16].

It has been shown that the combination of quercetin and naringenin synergistically regulates the expression of genes involved in antioxidant defense, including Nrf2, HO-1, SOD, CAT, and GPx [24]. Quercetin and rutin were found to prevent the DNA damage induced by the anticancer drug idarubicin by regulating the expression of genes involved in reductive bioactivation [25]. Cancer-related genes such as KRAS, MYC, CCND1, CDK6, BCL2, MCL1, BIRC5, XIAP, ZEB1, ZEB2, SNAIL1, SNAIL2, and VEGFA were shown to have their expression altered by quercetin and naringenin [26]. The expression of inflammatory and immunological genes such as NF-B, COX-2, iNOS, TNF, IL-6, and IL-8 was demonstrated to be regulated by quercetin, naringenin, and rutin [27].

In particular, quercetin has been demonstrated to activate p53, a transcription factor that plays a crucial role in regulating cell cycle progression and death [27]. A similar pattern of gene and transcription factor activation has been seen for naringenin, which has been linked to suppressing cell proliferation, angiogenesis, and inflammation. Some examples of such transcription factors are the tumor suppressor genes p53 and PTEN, and the inflammatory response regulator NF-κB [28]. There has not yet been a comprehensive investigation of quercetin, naringenin, and rutin’s positive benefits. Therefore, this study sets out to investigate if rutin, quercetin, and naringenin may serve as preventative medicine by influencing the body’s natural target network.

## 2. Results

The physicochemical properties of quercetin, rutin, and naringenin were evaluated with SwissADME. According to the analysis, all the studied drugs were predicted to be orally bioavailable, with rutin having the least bioavailability. All three flavonoids inhibited one or other cytochromes, and gastrointestinal absorption was high for quercetin and naringenin (Table 1).

**Figure 1:**
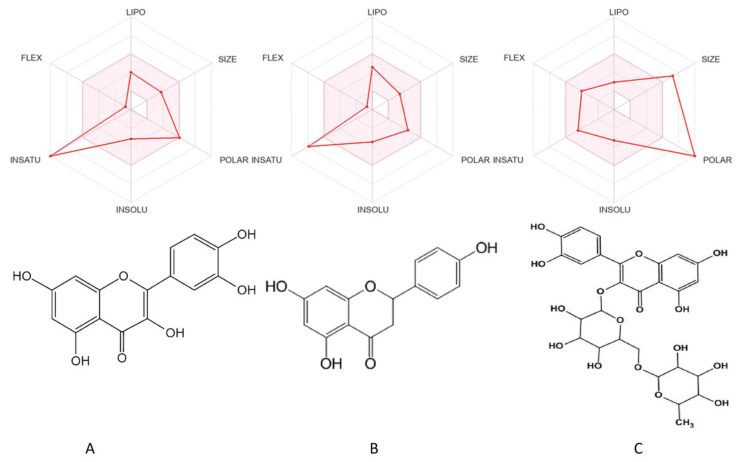
Illustrations of the structures of flavonoids and radar charts that represent their pharmacokinetic and physicochemical properties. The structures of (**A**) quercetin, (**B**) naringenin, and (**C**) rutin were adapted from SwissADME’s Marvin J S tool for visualization purposes in our study.

The structures of quercetin, naringenin, and rutin, along with their bioavailability radar plots for drug likeliness, are shown in Figure 1.

**Figure 2:**
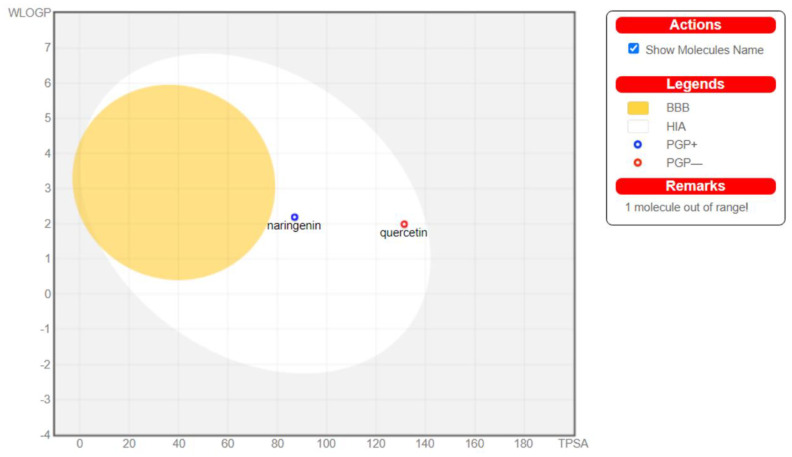
Boiled egg demonstration of quercetin, naringenin, and rutin.

Effective solubility was seen for all three studied drugs, with rutin being the most soluble of the three. Drug likeliness, as shown in radar plots (Figure 1), was higher for rutin than quercetin and naringenin. Passive human gastrointestinal absorption was high for quercetin and naringenin, whereas it was low for rutin, and all three did not cross the blood–brain barrier. Additionally, the results from the boiled egg graph (Figure 2) suggest that naringenin and quercetin were P-glycoprotein substrates, while rutin lied outside the range and was not the substrate for Pgp. Quercetin and naringenin were expected to be well absorbed by passive gastrointestinal absorption and to not cross the blood–brain barrier. The presence of a red dot in the egg white for quercetin demonstrates that it is a Pgp non-substrate, and a blue dot for naringenin demonstrates that it is a Pgp substrate and can be pumped out. Rutin was found to be out of the range between WLOGP and TPSA.

**Figure 3:**
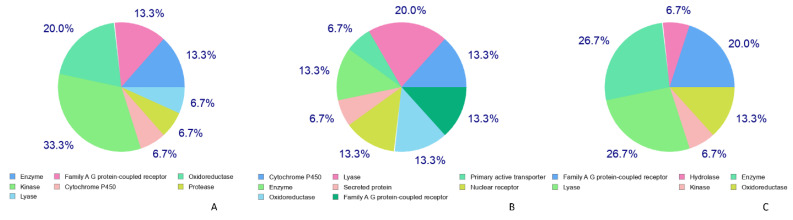
Target classes binding to quercetin, naringenin, and rutin using SwissADME’s target prediction tool. (**A**) Quercetin targets; (**B**) naringenin targets, and (**C**) rutin targets.

Figure 3 shows the targets of the respective flavonoid binding in cells to show their protective action. It was observed that quercetin binds mainly to kinases (33.3%), whereas both naringenin and rutin mainly bind to lyases, and the binding was observed to be 20% for either of them. Altogether, the main targets (up to 90%) of quercetin, naringenin, and rutin were enzymes, as shown in Figure 3.

**Figure 4:**
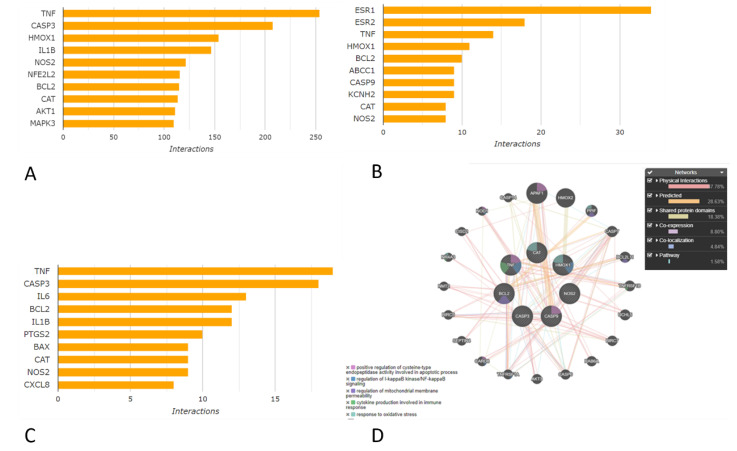
The top significantly interacting proteins with flavonoids. (**A**) quercetin protein interactions; (**B**) naringenin protein interactions; and (**C**) rutin protein interactions (**D**) The GeneMANIA predicted interaction of targeted genes with the quercetin, rutin and naringenin.

Further, we examined the direct flavonoid binding to functional units of genes, and our data mining found that quercetin, naringenin, and rutin show protective effects by regulating diverse genes. The common targets regulated were NOS2, CASP3, CASP9, CAT, BCL2, TNF, and HMOX1 (http://ctdbase.org/search/, accessed on 10 April 2023). As can be seen by Figure 4, the top-most interactions were shown by quercetin (approximately 250 interactions with TNF), followed by rutin (approximately 20 interactions), and naringenin (approximately 18 interactions with TNF). (D) The GeneMANIA tool was used to depict the gene interactions of the proteins obtained through the CTD tool. The possible benefits of protective effects were provided by 47.7% physically interacting genes, with 46% predicted and shared domains, and with an 8.8% co-expression of these genes.

The main genes interacting with quercetin are shown in Figure 4A. The genes interacting with naringenin are in Figure 4B, and the genes interacting with rutin in Figure 4C. The GeneMANIA prediction plug-in presented interaction types among the identified target genes for all the three drugs, as well as the possible benefits of protective effects by physically interacting with 47.7% of the other genes, with 46% predicted and shared domains, and with an 8.8% co-expression with genes (GeneMANIA, http://geneMANIA.org/plug-in/, accessed on 10 April 2023), as shown in Figure 4D.

**Figure 5:**
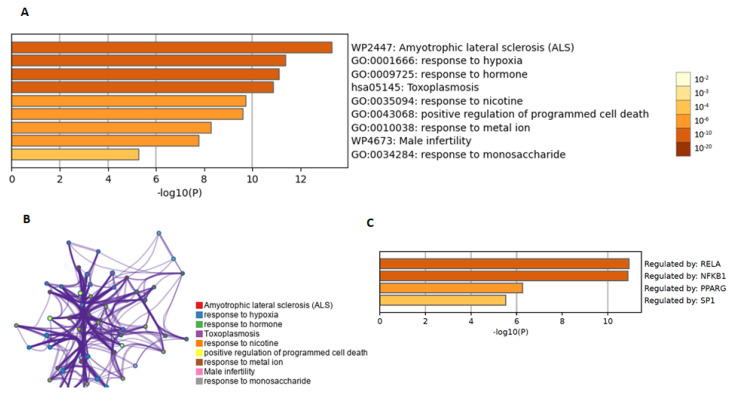
The statistically significant enriched terms, hierarchically clustered for the three flavonoids. (**A**) Biological processes or pathways and diseases that may be treated by the three flavonoids; and (**B**) the network clusters converted into a network layout that showed a similarity score greater than 0.3. The darker the color, the more statistically significant the node (**C**) Transcription factors involved in gene regulation.

Metascape (https://metascape.org/, accessed on 10 April 2023) and the Cytoscape plug-in ClueGO + CluePedia versions 2.5.8 and 1.5.8 were used to determine the enriched biological pathways and protein–protein interaction enrichment analyses, respectively. This was conducted for the analyzed genes with protective effects and disease protection that concerned the aforementioned common target genes. The most significant pathways associated with protective effects were those involving amyotrophic lateral sclerosis; responses to hypoxi; responses to monosaccharides, hormones, nicotine, and metal ions; toxoplasmosis; male infertility; and cell death (Figure 5A). Cytoscape version 3.9.1 was used to examine the protein–protein interactions of the seven aforementioned genes. Vertices and arcs are presented as clusters (Figure 5B).

**Figure 6:**
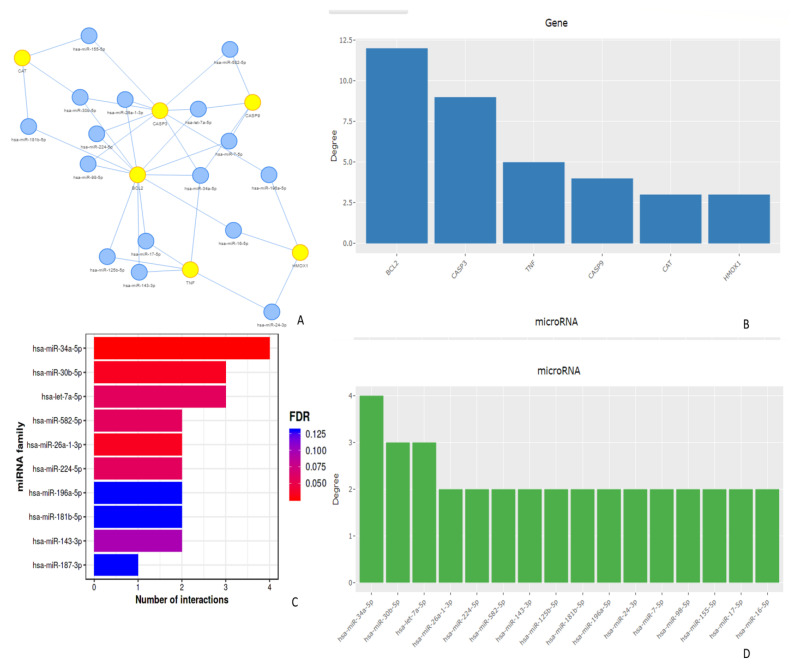
A network of the miRNA target interactions that were targeted by the three flavonoids via MIENTURNET. (**A**) Networks with microRNA and genes; (**B**) a bar illustrates the degree of involvement of each gene in protective mechanisms; (**C**) the number of interactions of each miRNA, (FDR means false discovery rate); and (**D**) the degree of involvement of each miRNA in protective mechanisms.

A GO enrichment analysis was applied to each MCODE network to extract the “biological meanings” from the network component, where the top three best *p*-value terms (namely, amyotrophic lateral sclerosis, epithelial cell apoptotic process, and response to hypoxia), were retained. The further flavonoid action through the genes NOS2, CASP3, CASP9, CAT, BCL2, TNF, and HMOX1, as well as through miRNAs was identified with the MIENTURNET web tool. The most important transcription factors that regulated these seven genes were RELA, NFKB1, PPARG, and SP1 (Figure 5C). The biological significance of the involved miRNAs was determined by constructing a miRNA target interaction network and conducting functional enrichment analyses. Specific miRNAs were associated with target genes. We identified the 20 microRNAs (miRNAs) involved in regulating the above mentioned seven gene targets (Figure 6A). The degree of miRNA interactions was obtained with a maximum degree in the BCl-2 gene, followed by CASP3, TNF, CASP9, CAT, and HMOX1 (Figure 6B).

Quercetin, naringenin, and rutin are examples of caspase inhibitors, suggesting that Bcl-2 regulates these proteins. This inhibition is achieved by significantly increasing the expression and interaction of specific microRNAs (hsa-miR-34a-5p, hsa-miR-30b-5p, hsa-let-7a-5p, and hsa-miR-26a-1-3p). Among these microRNAs, hsa-miR-34a-5p demonstrated the highest number of interactions, followed by hsa-miR-30b-5p, hsa-let-7a-5p, and hsa-miR-26a-1-3p (Figure 6D). These miRNAs have been identified as tumor suppressors in several cancer types, and they play a critical role in regulating processes relevant to cancer, including proliferation, apoptosis, epithelial–mesenchymal transition, metastasis, and the activity of endothelial nitric oxide synthase.

## 3. Discussion

Flavonoids are antioxidants, and they have anti-inflammatory, anti-hypoxia, and anti-apoptotic properties; furthermore, they also reduce metabolic disorders [29,30,31]. They can modulate these processes by affecting the activity and expression of enzymes and genes. Polyphenols are powerful antioxidants that can initiate anti-oxidative stress and anti-inflammatory pathways [32]. Anti-inflammatory enzymes such as lysozyme and beta-glucuronidase are inhibited, as is arachidonic acid production. These can inhibit the production and activation of important mediators of inflammatory reactions, such as IL-1, TNF-, IL-6, and IL-8. By modulating the expression and activity of transcription factors such as NF-kB, AP-1, ICAM, VCAM, and E-selectins (which govern the synthesis of various pro-inflammatory chemicals), flavonoids can further modulate inflammation. By decreasing the expression and activity of pro-inflammatory enzymes such as inducible nitric oxide synthase, cyclooxygenase-2 (COX-2), and lipoxygenase (LOX), flavonoids can limit the generation of inflammatory mediators such as nitric oxide (NO), prostaglandins (PGs), and leukotrienes (LTs) [33,34].

In addition, flavonoids can exert an effect on the expression, as well as on the activity of apoptotic proteins such as BCL-2, BAX, and cleaved caspase-3 (CASP3), which ultimately leads to the induction of apoptosis in cancer cells. Polyphenols are found in a variety of plant-based foods and beverages [33,35]. They can influence the process of PI3K/AKT/mTOR signaling, which is necessary for the survival of cells, the proliferation of cells, the metabolism of cells, and angiogenesis. They can also raise the level of the phosphorylation of AKT and mTOR, which results in an increase in cell survival and a suppression of apoptosis [35]. They alter both glucose and lipid metabolism [36], which is likely due to their capacity to control the expression and activity of enzymes such as AMPK, SIRT1, PGC-1, and PPAR.

However, despite the extensive range of protective effects exhibited by flavonoids, further research is necessary to gain a comprehensive understanding of the underlying molecular mechanisms. To determine which genes, transcription factors, and microRNAs (miRNAs) contribute to quercetin, naringenin, and rutin’s protective properties, computational analysis was used in this work. The studied drugs of quercetin, naringenin, and rutin showed promise for oral bioavailability, and enzymes including kinases and lyases were shown to be their principal targets of activity. The genes NOS2, CASP3, CASP9, CAT, BCL2, TNF, and HMOX1 were shown to be targets in a large number of different studies. The identification of these genes implicated in flavonoid activity will assist in the elucidation of protective pathways, paving the way for novel treatment approaches to a wide range of illnesses [37]. Previous studies have also reported that the neuroprotective effect involves the induction of Bcl-2 and Bcl-x expression through the activation of TNF (tumor necrosis factor) [28]. Caspase-9 (CASP9) and CASP3 were additional important factors in the protective effect. CASP9 is essential for the homeostasis of cells because it facilitates the cleavage of many of the critical factors involved in apoptosis. This is because apoptosis is vital for maintaining the homeostasis of the cell. CASP9 is essential for eliminating cells by executing apoptotic death early in the development stage. It is also indispensable for inhibiting proliferative diseases through the continuous removal of irreparable cells in the lifecycle and activation of CASP3 [38]. CASP9 is necessary for the constant elimination of damaged cells throughout a lifetime, which is necessary to limit the progression of proliferative disorders. Similarly, it has been discovered that the regulation of inducible nitric oxide synthase and CAT expression plays an important role in inflammation management, infection control, and immunological modulation.

The pharmacological regulation of these enzymes involved in the generation of mononuclear phagocytes is likely a useful therapeutic strategy [39]. Furthermore, catalase helps buffer free NO when potentially toxic concentrations of NO are approached [40].

The protective effects of flavonoids include amyotrophic lateral sclerosis, the hypoxic response, the monosaccharide response, hormone regulation, nicotine and metal ion toxicity, toxoplasmosis, male infertility, and cell death. They have a protective effect on the motor neurons against oxidative stress and inflammation, which are significant contributors to the initiation and progression of amyotrophic lateral sclerosis [41]. Quercetin, naringenin, and rutin have shown promise for enhancing oxygen delivery to tissues, reducing oxidative stress, and stimulating mitochondrial function; hence, providing potential therapeutic benefits for hypoxia-related diseases [42,43]. They have been discovered to alter the response to monosaccharides, suggesting that they may be useful in the prevention and treatment of diabetes and other metabolic disorders by regulating glucose metabolism, increasing insulin sensitivity, and lowering inflammation [44,45].

Moreover, they affect hormone control and hormone receptor function, as well as suppress hormone-related cancer risk [46,47]. They reduce the cell and tissue damage caused by nicotine and metal ions because of their protective qualities [48,49]. According to studies, flavonoids limit the development of Toxoplasma gondii, the parasite that causes toxoplasmosis, and boost the immunological response to infection [50]. In addition, flavonoids have shown promise in enhancing sperm quality, decreasing DNA damage, and encouraging cell survival, hence providing potential advantages for male infertility [51,52].

Quercetin, naringenin, and rutin significantly increase the expression and interaction of the tumor suppressor microRNA hsa-miR-34a-5p, which is linked to various cancers. Cell proliferation, apoptosis, epithelial–mesenchymal transition (EMT), and metastasis are only some of the cancer-related activities that this microRNA controls [53,54]. The miR-153 gene, identified as a significant factor, has demonstrated tumor suppressive effects across various cancer types. It works by controlling the expression of several genes that play crucial roles in the body. Notably, miR-153 is believed to modulate the activity of oncogenes, including KRAS, MYC, CCND1, and CDK6. It also affects the proteins involved in preventing cell death, called anti-apoptotic proteins, such as BCL2, MCL1, BIRC5, and XIAP. Furthermore, miR-153, which is involved in EMT and metastasis, regulates transcription factors such as ZEB1, ZEB2, SNAIL1, and SNAIL2. In addition, miR-153 regulates the production of VEGFA (growth factor) [31]. By regulating these genes, miR-153 efficiently slows cancer cell growth, causes apoptosis, suppresses EMT, and impedes metastasis. Due to its properties, miR-153 might be used as a therapeutic agent and biomarker in cancer diagnosis and therapy.

**Figure 7:**
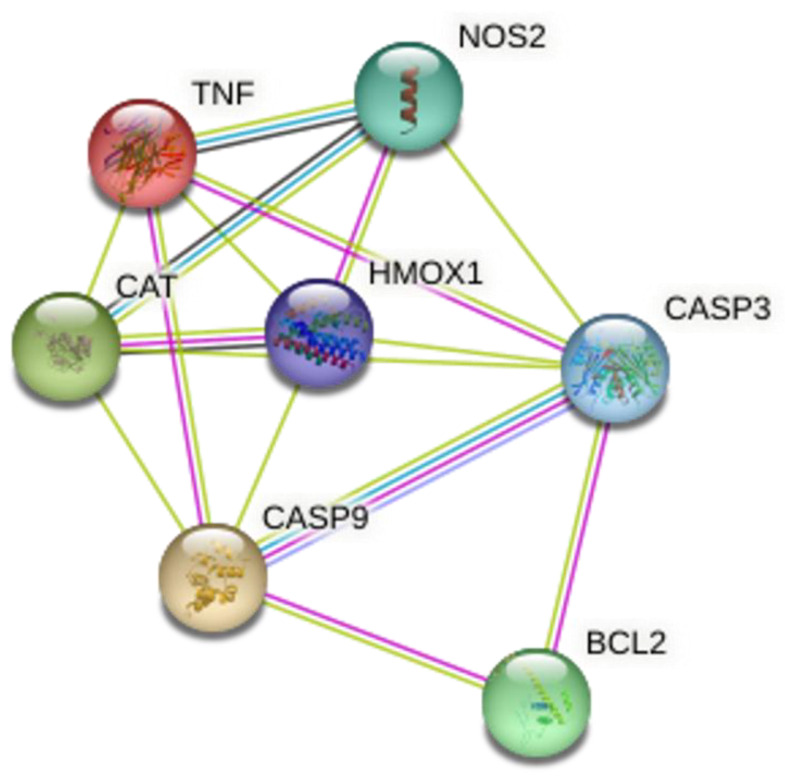
The major contributor genes involved in flavonoid interaction.

The apoptosis regulator Bcl-2 is in concert among the target genes of quercetin, naringenin, and rutin, as revealed by the STRING and PPI enrichment analysis of Bcl-2, which has been demonstrated to inhibit apoptosis in several different cell types, including those dependent on extracellular stimuli such as lymphohematopoietic and central nervous system cells. It accomplishes this by controlling the permeability of the mitochondrial membrane, which leads to cell death. Bcl-2 also interacts with caspases in a feedback loop. It inhibits caspase activity via attaching to the apoptosis-activating factor or by blocking the release of mitochondrial cytochrome c. (APAF-1). Apoptosis is carried out through a caspase activation cascade. CASP9 activates, leading to the cleavage and activation of CASP3, upon binding to Apaf-1. DNA damage induces apoptosis by this pathway, which leads to the proteolytic cleavage of poly (ADP-ribose) polymerase (PARP) and the formation of the apoptosome. CASP3, CASP9, tumor necrosis factor (TNF), and Bcl-2 were shown to be major contributory genes in connection with flavonoid action, amyotrophic lateral sclerosis (ALS), the epithelial cell apoptotic process, and hypoxia (Figure 7).

## 4. Methods

### 4.1. Pharmacokinetics and Physicochemical Characteristics

The pharmacokinetic and physicochemical properties of quercetin, naringenin, and rutin were predicted using SwissADME. SwissADME was able to provide predictions about the pharmacokinetic factors such as solubility and lipophilicity, as well as absorption, distribution, metabolism, and excretion (ADME). SwissADME (http://www.swissadme.ch/, accessed on 10 April 2023) employs many algorithms and models through which to provide predictions about toxicity and drug-likeness. The algorithms used to generate the bioavailability radar chart are based on machine learning and statistical methods, and they are trained via large datasets of molecules with known properties. ADME properties were either positively or negatively detected in the BOILED-Egg model; these included passive human gastrointestinal absorption (HIA), blood–brain barrier (BBB) permeation, and the substrate or non-substrate of the permeability glycoprotein (P-gp) [55,56,57]. Additionally, the Swiss target prediction tool was utilized to anticipate the direct and indirect target genes and proteins.

### 4.2. Gene Association Network Analysis

By focusing on the SwissADME-obtained common target genes for the three flavonoid drugs, we analyzed the interaction network with the associated genes. We used the GeneMANIA (https://genemania.org/, accessed on 10 April 2023) program to examine the collection of genes that mediated the impact of these flavonoids with respect to physical interaction, genetic interaction, co-expression, co-localization, shared protein domains, and anticipated relationships. GeneMANIA is a web-based tool used for predicting functional relationships among genes. It is a powerful gene network analysis platform that integrates diverse functional genomics datasets to generate hypotheses about gene function, including gene co-expression, genetic interactions, protein–protein interactions, pathways, and functional annotations. The gene set obtained was further refined by the Comparative Toxicogenomics Database (CTD) (http://ctdbase.org/search/, accessed on 10 April 2023). CTD showed that, in total, there were seven genes (NOS2, CASP3, CASP9, CAT, BCL2, TNF, and HMOX1) that mostly interacted with the three flavonoids. The same genes were found in the target gene list obtained from SwissADME. Further, each flavonoid was analyzed for its relationship with disease protection by choosing animal disease as an option and by filtering for all. The associated genes were analyzed for involvement in the biological processes [58].

### 4.3. Enrichment Analysis

To better comprehend the pathways and diseases on which these polyphenols have a protective effect, an enrichment analysis of the gene list was performed using Metascape. This allowed us to identify biological pathways and protein–protein interaction enrichments. Metascape automatically converted the EggNOG and Homologene databases into Human Entrez Gene IDs for NOS2, CASP3, CASP9, CAT, BCL2, TNF, and HMOX1. Accumulative hypergeometric distribution values were used to calculate the *p*-values, the Benjamini–Hochberg process was used to generate q-values, and enrichment factors were computed and used as filters to obtain datasets. Metascape provides a platform for developers to create plugins and extensions to extend the functionality of the software. This has led to a rich ecosystem of plugins that can perform various network analysis tasks, such as community detection, pathway enrichment analysis, and network clustering. Protein–protein interaction (PPI) enrichment was investigated with STRING (version 11.5, https://string-db.org/, accessed on 10 April 2023) and Metascape (https://metascape.org/, accessed on 10 April 2023). Only physical interactions were evaluated in STRING (physical score > 0.132). Settings of a node density cutoff = 0:1, node score cutoff = 0:2, degree cutoff = 2, maximal depth = 100, and K-core = 2 were used to analyze the PPI network, with loops and fluff eliminated. In networks that had between 3 and 500 proteins, the molecular complex detection (MCODE) approach was employed to find the strongly related network components [59].

### 4.4. miRNA Target Interactions

A microRNA-enrichment-turned network (MIENTURNET; Rome, Italy; http://userver.bio.uniroma1.it/apps/mienturnet/, accessed on 10 April 2023) was used to generate and analyze the miRNA-target interaction networks. Data from the genes NOS2, CASP3, CASP9, CAT, BCL2, TNF, and HMOX1 were predicted using MIENTURNET for this study. To create miRNA networks, we turned to MiRTarBase. Functional enrichment analysis was performed by scouring the Wikipathways and Disease Ontology databases with the help of the MIENTURNET web tool. Through using a 0.05 cutoff and the Benjamini–Hochberg method to adjust *p*-values, we analyzed functional annotations that were significantly enriched over the entire gene list in the input list [60].

## 5. Conclusions

The major findings of this study are that quercetin, rutin, and naringenin exhibit oral bioavailability and primarily target enzymes such as kinases and lyases. Many genes, including NOS2, CASP3, CASP9, CAT, BCL2, TNF, and HMOX1, are targeted by quercetin, naringenin, and rutin. The physicochemical parameters and pharmacokinetics of quercetin, rutin, and naringenin were evaluated to support their potential as therapeutic agents. Pathways associated with their protective effects, encompassing conditions such as amyotrophic lateral sclerosis, hypoxia response, monosaccharide response, hormonal regulation, nicotine and metal ion toxicity, toxoplasmosis, male infertility, and cell death, were identified. Twenty microRNAs (miRNAs) and four transcription factors were identified in our study as regulators of the gene targets, which may help explain the protective effects of these polyphenols. However, further research is needed, including prospective studies and clinical trials, before these flavonoids may be used to treat the conditions we identified.

## Figures and Tables

**Table 1:** Physicochemical properties for drug-likeness and physicochemical inspections of quercetin, rutin, and naringenin.

NAME	Quercetin	Naringenin	Rutin
Molecular weight	302.24	272.25	610.52
Num. heavy atoms	22	20	43
Num. aromatic heavy atoms	16	12	16
Fraction Csp3	0	0.13	0.44
Num. rotatable bonds	1	1	6
Num. H-bond acceptors	7	5	16
Num. H-bond donors	5	3	10
Molar refractivity	78.03	71.57	141.38
TPSA	131.36	86.99	269.43
Consensus Log Po/w	1.23	1.84	−1.29
ESOL Log S	−3.16	−3.49	−3.3
ESOL solubility (mg/mL)	0.211	0.0874	0.308
ESOL solubility (mol/l)	0.000698	0.000321	0.000505
ESOL class	Soluble	Soluble	Soluble
GI absorption	High	High	Low
BBB permeant	No	No	No
Pgp substrate	No	Yes	Yes
CYP1A2 inhibitor	Yes	Yes	No
CYP2C19 inhibitor	No	No	No
CYP2C9 inhibitor	No	No	No
CYP2D6 inhibitor	Yes	No	No
CYP3A4 inhibitor	Yes	Yes	No
Log Kp (cm/s)	−7.05	−6.17	−10.26
Lipinski violations	0	0	3
Ghose violations	0	0	4
Veber violations	0	0	1
Egan violations	0	0	1
Muegge violations	0	0	4
Bioavailability score	0.55	0.55	0.17
PAINS alerts	1	0	1
Brenk alerts	1	0	1
Lead-likeness violations	0	0	1
Synthetic accessibility	3.23	3.01	6.52

## Data Availability

Data will be available on request to the corresponding author.
